# CD117 expression predicted FLT3 mutation in T‐cell acute lymphoblastic leukemia

**DOI:** 10.1002/jha2.862

**Published:** 2024-02-18

**Authors:** Haijin Wang, Runfeng Ni, Aidi Wang, Baoshan Liu

**Affiliations:** ^1^ Department of Traditional Chinese Medicine Tianjin Medical University General Hospital Tianjin China; ^2^ Shanghai Municipal Hospital of Traditional Chinese Medicine Shanghai China

Dear Editor,

T‐cell acute lymphoblastic leukemia (T‐ALL) is a clonal hematopoietic stem cell disorder of T‐cell origin. T‐ALL, a common pediatric malignancy, accounts for approximately 15% of childhood ALL cases [1]. CD117 (KIT) is a type III receptor tyrosine kinase naturally expressing in various cell types, such as hematopoietic stem cells, germ cells, and melanocytes [2]. CD117 is detected at granulopoiesis until promyelocyte, so it is usually used to diagnose acute myelogenous leukemia. Moreover, CD117 is not normally expressed on T cells, so its expression indicates an aberrant immunophenotype, and some studies have suggested that it could be correlated with the FMS‐like tyrosine kinase 3 (FLT3) gene mutation. FLT3 gene mutations (FMS) are widely studied in blood diseases and occur in a variety of myeloid tumors, accounting for about 1% of ALL. About 5‐10% in MDS and 15‐35% in AML. The most common mutations are ITD (internal tandem repeat) and TKD (point mutation in the activation loop). A previous study reported FLT3 mutations only in CD117^+^ T‐ALL and suggested that CD117 expression might identify a subset of T‐ALL cases harboring FLT3 mutation [3]. However, the relevance of CD117 and FLT3 lacks cases and remains controversial. By searching databases, we conducted a retrospective study of the genes and immunophenotypes of patients diagnosed with T‐ALL or early T‐cell precursor acute lymphoblastic leukemia (ETP‐ALL). Our aim was to evaluate the expression of CD117 on T‐cell neoplasms for diagnostic and therapeutic utility.

We collected 323 eligible cases in eight relevant randomized controlled trials through database search from 2004 to 2023, as shown in Table S1. CD117 positivity was detected in 66 (20.43%) of the 323 samples analyzed. Interestingly, FLT3 gene mutations were detected in 23 (34.84%) of the CD117‐positive cases. The FLT3 mutation was mainly ITD, and other mutations, such as TKD and other, were not reported.

CD117 is co‐expressed with CD135, also known as FLT3 receptor in early thymic progenitors before T‐cell receptor gene rearrangement [4]. An analysis further revealed that most of the CD34 FLT3^+^ cells co‐express CD117 at a high level [5]. Moreover, FLT3 signaling contributed to early thymopoiesis by maintenance of ETP cellularity in physiological status [6]. Therefore, we are not surprised at detecting of 65.52% ETP‐ALL patients with CD117 positive, and in two studies, a total of eight samples (21.05%) showed FLT3 mutation. Importantly, the co‐expression was more obvious in T‐ALL; specifically, there are 41.67% FLT3‐mut CD117^+^ patients.

CD117 and FLT3, the hematopoietic growth factor receptors, are members of the same family of tyrosine kinases and crucial mediators of hematopoietic stem cell and common lymphoid progenitor cell proliferation and survival. Their mutations or alternations result in aberrant signaling, which is a crucial driving force in tumorigenesis. Both KIT and FLT3 regulate the MAPK (mitogen‐activated protein kinase, MAPK), PI3K (Phosphatidylinositol‐3‐hydroxykinase, PI3K), and JAK/STAT (Janus tyrosine kinase/signal transducer and activator of transscription, JAK/STAT) pathway to affect the proliferation, survival, and transformation of hematopoietic cells, resulting in myelodysplastic syndrome and acute leukemia [7]. Therefore, CD117 and FLT3 could have common mechanism in the pathogenesis and progress of T‐ALL.

CD117 is considered as a marker to predict FLT3 expression or mutation and guiding treatment; however, a single immunophenotype is less convincing. Hoehn et al. suggested that the immunophenotypic profile of TdT, CD7, CD13, CD34, and CD117 (bright) is helpful for predicting FLT3 mutation, with a sensitivity of 100% and specificity of 94% [8]. Another study demonstrated that a unique immunophenotype panel is a better predictor [9]. Overall, the immunophenotype, including CD117, could recognize and predict FLT3 mutation. Furthermore, previous studies have shown that inhibition of KIT and FLT3 effectively suppressed the proliferation of tumor cells from patients with T‐ALL [10]. Above all proved that CD117 could predict FLT3 mutation and had synergistic treatment effect in T‐ALL.

In short, CD117 expression is associated with FLT3 expression or mutation. A more precise immunophenotypic profile including CD117 may be a substitute for predicting FLT3 expression or mutation in T‐ALL. We need to carefully recognize FLT3‐mut CD117^+^ T‐ALL in clinical work and get clinical date for exploring more effective therapy, such as KIT or FLT3 inhibitor therapy.

## AUTHOR CONTRIBUTIONS

Runfeng Ni and Haijin Wang made substantial contributions to conception and design, acquisition of data, analysis, and interpretation of the data, as well as drafting the article or revising it critically for important intellectual content. Aidi Wang and Baoshan Liu made substantial contributions to conception and design, acquisition of data, analysis, and interpretation of data.

## CONFLICT OF INTEREST STATEMENT

The authors declare they have no conflicts of interest.

## ETHICS STATEMENT

The authors have confirmed ethical approval statement is not needed for this submission.

## PATIENT CONSENT STATEMENT

The authors have confirmed patient consent statement is not needed for this submission.

## PERMISSION TO REPRODUCE MATERIAL FROM OTHER SOURCES

The article did not reproduce material from other sources.

## CLINICAL TRIAL REGISTRATION (INCLUDING TRIAL NUMBER)

The authors have confirmed clinical trial registration is not needed for this submission.

## Supporting information

Supporting Information

Supporting Information

## Data Availability

Not applicable.
