# Supplementary material for: CD117 expression predicted FLT3 mutation in T‐cell acute lymphoblastic leukemia
Source: EJHaem. 2024 Feb 18;5(1):294–5. doi: 10.1002/jha2.862 (PMC10887319; doi:10.1002/jha2.862)
Supplement: Supplementary file 1 — Supporting Information [file JHA2-5-294-s002.docx]

By searching English databases PubMed, Web of Science, Cochrane Central Register of Controlled Trials, Cochrane Database of Systematic Reviews, Springer-Link, Wiley and Chinese databases China National Knowledge Infrastructure (CNKI), Wanfang Database, using "FLT3" and "CD117" and "T-ALL" as keywords, found 8 relevant articles from 2004 to 2023, and a total of 323 cases of T-ALL and ETP-ALL patients and summarized 66 cases expressing CD117, of which 23 cases carried the FLT3 mutation.

**General sample characteristics according to the neoplasms**

| **Published years, study and reference** | **Population** | **Type of disease** | **Expression of immunophenotype** | **Gene mutations** |
| --- | --- | --- | --- | --- |
| 2023, Ye et al. ^[1]^ | 32 | ETP-ALL | CD117  (21/32, 68%) | FLT3  (4/21, 19%) |
| 2012, Hoehn et al.^[2]^ | 27 | T-ALL | CD117  (11/27, 41%) | FLT3-ITD  (1/11, 19%) |
| 2011, Heesch, et al.^[3]^ | 38 | T-ALL | CD117  (9/38, 24%) | FLT3  (3/7, 43%) |
| 2004, Paietta et al.^[4]^ | 69 | T-ALL | CD117  (3/69, 4%) | FLT3-ITD  (3/3, 100%) |
| 2005, Van Vlierberghe et al.^[5]^ | 72 | T-ALL | CD117  (2/72, 2.7%) | FLT3-ITD  (2/2, 100%) |
| 2019, Noronha et al.^[6]^ | 26 | ETP-ALL | CD117  (17/26, 65%) | FLT3-ITD  (4/17, 23%) |
| 2012, Zaremba et al.^[7]^ | 38 | T-ALL | CD117  (7/38, 24%) | FLT3-ITD  (3/7, 43%) |
| 2020, Shen et al.^[8]^ | 21 | T-ALL | CD117  (6/21, 28%) | FLT3-ITD  (3/6, 50%) |

**Reference:**

[1] YE M T, WANG Y, ZUO Z, et al. Integrated clinical genotype-phenotype characteristics of early T-cell precursor acute lymphoblastic leukemia [J]. Cancer, 2023, 129(1): 49-59.<http://dx.doi.org/10.1002/cncr.34515>

[2] HOEHN D, MEDEIROS L J, CHEN S S, et al. CD117 expression is a sensitive but nonspecific predictor of FLT3 mutation in T acute lymphoblastic leukemia and T/myeloid acute leukemia [J]. Am J Clin Pathol, 2012, 137(2): 213-9.<http://dx.doi.org/10.1309/AJCPR3N3JMSYLPFG>

[3] HEESCH S, BARTRAM I, NEUMANN M, et al. Expression of IGFBP7 in acute leukemia is regulated by DNA methylation [J]. Cancer Sci, 2011, 102(1): 253-9.<http://dx.doi.org/10.1111/j.1349-7006.2010.01760.x>

[4] PAIETTA E, FERRANDO A A, NEUBERG D, et al. Activating FLT3 mutations in CD117/KIT(+) T-cell acute lymphoblastic leukemias [J]. Blood, 2004, 104(2): 558-60.<http://dx.doi.org/10.1182/blood-2004-01-0168>

[5] VAN VLIERBERGHE P, MEIJERINK J P, STAM R W, et al. Activating FLT3 mutations in CD4+/CD8- pediatric T-cell acute lymphoblastic leukemias [J]. Blood, 2005, 106(13): 4414-5.<http://dx.doi.org/10.1182/blood-2005-06-2267>

[6] NORONHA E P, MARQUES L V C, ANDRADE F G, et al. T-lymphoid/myeloid mixed phenotype acute leukemia and early T-cell precursor lymphoblastic leukemia similarities with NOTCH1 mutation as a good prognostic factor [J]. Cancer Manag Res, 2019, 11(3933-43.<http://dx.doi.org/10.2147/CMAR.S196574>

[7] ZAREMBA C M, OLIVER D, CAVALIER M, et al. Distinct immunophenotype of early T-cell progenitors in T lymphoblastic leukemia/lymphoma may predict FMS-like tyrosine kinase 3 mutations [J]. Ann Diagn Pathol, 2012, 16(1): 16-20.<http://dx.doi.org/10.1016/j.anndiagpath.2011.07.005>

[8] SHEN Z, CHU X L, WANG R X, et al. The Clinical and Molecular Characteristics of FLT3 Mutations in Chinese De Novo Adolescent and Adult Acute Lymphoblastic Leukemia Patients [J]. Clin Lymphoma Myeloma Leuk, 2020, 20(6): e259-e69.<http://dx.doi.org/10.1016/j.clml.2019.09.602>
